# Myocardial capacity of mitochondrial oxidative phosphorylation in response to prolonged electromagnetic stress

**DOI:** 10.3389/fcvm.2023.1205893

**Published:** 2023-06-07

**Authors:** Lesia Savchenko, Ilenia Martinelli, Dimitri Marsal, Vyacheslav Zhdan, Junwu Tao, Oksana Kunduzova

**Affiliations:** ^1^ National Institute of Health and Medical Research (INSERM) U1297, Toulouse, France; ^2^ Toulouse University, Toulouse, Cedex 9, France; ^3^ Poltava State Medical University, Poltava, Ukraine; ^4^ Toulouse, INP-ENSEEIHT, LAPLACE, Toulouse, France

**Keywords:** electromagnetic stress, heart, metabolism, oxidative phosphorylation, superoxide dismutase

## Abstract

**Introduction:**

Mitochondria are central energy generators for the heart, producing adenosine triphosphate (ATP) through the oxidative phosphorylation (OXPHOS) system. However, mitochondria also guide critical cell decisions and responses to the environmental stressors.

**Methods:**

This study evaluated whether prolonged electromagnetic stress affects the mitochondrial OXPHOS system and structural modifications of the myocardium. To induce prolonged electromagnetic stress, mice were exposed to 915 MHz electromagnetic fields (EMFs) for 28 days.

**Results:**

Analysis of mitochondrial OXPHOS capacity in EMF-exposed mice pointed to a significant increase in cardiac protein expression of the Complex I, II, III and IV subunits, while expression level of α-subunit of ATP synthase (Complex V) was stable among groups. Furthermore, measurement of respiratory function in isolated cardiac mitochondria using the Seahorse XF24 analyzer demonstrated that prolonged electromagnetic stress modifies the mitochondrial respiratory capacity. However, the plasma level of malondialdehyde, an indicator of oxidative stress, and myocardial expression of mitochondria-resident antioxidant enzyme superoxide dismutase 2 remained unchanged in EMF-exposed mice as compared to controls. At the structural and functional state of left ventricles, no abnormalities were identified in the heart of mice subjected to electromagnetic stress.

**Discussion:**

Taken together, these data suggest that prolonged exposure to EMFs could affect mitochondrial oxidative metabolism through modulating cardiac OXPHOS system.

## 1. Introduction

Mitochondria are exuberantly present in cardiac muscle and serve as the capital source of energy production. In cardiac cells, the primary function of the mitochondria is to federate the high energy requirement of the beating heart by generating energy in the form of adenosine triphosphate (ATP) through oxidative phosphorylation (OXPHOS) machinery (1, 2). The OXPHOS system requires an orchestrated transfer of electrons via five multi-subunit enzymes: Complex I (NADH dehydrogenase or NADH: ubiquinone oxidoreductase), Complex II (succinate dehydrogenase or succinate:ubiquinone oxidoreductase), Complex III (the bc1 complex or ubiquinone: cytochrome c oxidoreductase), Complex IV (cytochrome c oxidase, cyclooxygenase or reduced cytochrome c: oxygen oxidoreductase), and Complex V (ATP synthase), which are localized on the inner mitochondrial membrane (3–5). Furthermore, OXPHOS machinery also comprises two electron transport carriers, ubiquinone or coenzyme Q10 and cytochrome c (6, 7). In the course of electron transport, Complexes I, III, and IV affiliate protons from the mitochondrial matrix to the intermembrane space culminating in enhanced membrane potential (8). In the presence of adenosine diphosphate (ADP), Complex V favors flow of protons to the matrix culminating in ATP formation (3, 4). Mitochondrial OXPHOS complexes are not only responsible for energy generation but also connected to the multifaceted cell events, including reactive oxygen species (ROS) formation, inflammation, and apoptosis (9, 10). Therefore, the defects in the OXPHOS system, originating from genetic or environmental factors, have been reported in multiple diseases including heart failure, arrhythmia, and hypertension (10).

Electromagnetic fields (EMFs) depict one of the most prevalent growing environmental counterinfluences on biological systems. The exceeding common exposure to EMFs occurs from telecommunications networks, public broadcast infrastructure, wireless technologies such as Wi-Fi, and the operation of mobile devices. Several sources of EMFs are also used in medical and industrial applications and security scanning equipment. Therefore, exposure to EMFs, particularly those emitted by mobile communications transmitting at 900 MHz, remain a much controversial topic, which is related to their potential effects on human health. Both elevated cardiovascular risk and marked cardioprotection have been attributed to EMF exposure (11, 12). It has been reported that these effects are mediated by mitochondrial ROS, which are involved in vital cell decisions and can be capital or excessively toxic to cellular homeostasis (13). To alleviate ROS-mediated cellular damage, living organisms have anti-oxidative mechanisms including superoxide dismutase (SOD), glutathione peroxidase, and catalase (14). Previous research has mainly concerned the short-term biological effects of EMFs on oxidative stress and antioxidant mechanisms; however, long-term impact of EMFs has been a relatively minor focus.

Considering the central place of mitochondrial metabolism in the acquisition and maintenance of cardiac cell integrity, in this study, we examined the prolonged exposure to 915 MHz EMFs on mitochondrial respiratory function and oxidative stress status in relation to structural integrity of the myocardium.

## 2. Materials and methods

### 2.1. Animal studies

Animal experiments were performed in accordance with the guidelines established by the European Communities Council Directive (2010/63/EU Council Directive Decree) and approved by the local ethical committee (project N 20/1048/14/02, national agreement 2020072717253128). Adult Swiss Webster male mice 9 months old were used for these investigations (Envigo RMS, France). Animals were given free access to standard food and water under controlled conditions. Two days prior to experiments, mice were adapted to new environmental conditions (light was on a 12 h light–12 h dark cycle) as previously described (15). Mobile communication technology operates at 900 MHz frequency; thus, we focus on the effects of frequency close to 900 MHz. Mice were randomly divided into two groups: control sham (*n* = 7) and EMFs (*n* = 8) groups, respectively. The mice from EMF group were exposed to a 915 MHz EMFs for 28 days (9 h/day). Control groups of animals did not exposure of EMF. Animals were sacrificed and hearts were removed and rinsed in 4°C phosphate-buffered saline (PBS). Heart weight and body weight were measured the day of sacrifice. Heart weight to body weight ratio in grams was calculated by dividing the weight of the heart by the weight of the whole animal. Harvested tissues were divided into three parts: (1) embedded into optimal cutting temperature compound (OCT) (Sigma-Aldrich, Saint-Quentin-Fallavier, France) under ice-cold 2-methylbutane for cryo-section, (2) for Western blot, and (3) the remaining portion was used for quantitative polymerase chain reaction (qRT-PCR).

### 2.2. Radio frequency approach to an *in vivo* model

To induce electromagnetic stress, *in vivo* experiments were performed in a Giga-TEM (GTEM) cell. For animal adaptation, 24 h prior to tests mice were placed in the GTEM cell. A solid-state radiofrequency generator with a fixed frequency of 915 MHz (WSPS-915–1000) (Chengdu Wattsine Electronics Technology, Chengdu, Sichuan, China) was used for *in vivo* experiments. The Webster was modeled by the sphere of equivalent volume in these simulations. Three spheres were implanted along the wave propagation axis of the GTEM cell. The relative permittivity value was 55, the conductivity of 3 S/m were applied for the experimental protocol. The estimated specific absorption rate (SAR) values were around 40 W/kg. Using MATLAB codes, the calculation of whole body SAR was taken by volume integration of absorption power as previously described (15, 16). In radio frequency (RF) testing, the input power was 4 W in the high frequency structure simulator (HFSS) simulation and in the experimentation, as previously described (15, 16).

### 2.3. Western blot

Extraction of proteins from cardiac tissues was performed as previously described (15, 17). Briefly, for protein extraction, the RIPA buffer (50 mM Tris–HCl, 150 mM NaCl, 0.1% SDS, 0.5% sodium deoxycholate, 1% Triton X-100, 1 mM EDTA, completed with protease and phosphatase inhibitor cocktails) was used. To determine the proteins concentration of the lysate, we used the Bio-Rad Protein Assay (Bio-Rad, Hercules, CA, United States). Equivalent protein amounts were loaded in each lane of gels. Proteins were loaded in the Laemmli sample buffer, denaturated, and were resolved by sodium dodecyl sulfate-polyacrylamide gel electrophoresis (SDS-PAGE) and Western blotting. Furthermore, the proteins were separated by electrophoresis and transferred to a nitrocellulose membrane (Amersham Protran, GE Healthcare, Germany) using electroblotting apparatus (Bio-Rad, Hercules, CA, United States). Then, membranes were incubated with 5% bovine serum albumin (BSA) in Tris-buffered saline tween-20 buffer (TBST; 25 mM Tris, pH 7.5, 150 mM NaCl, and 0.1% Tween20) for 1 h to prevent nonspeciﬁc binding sites and incubated overnight at 4°C with the primary antibodies. In final steps, immunoreactive bands were detected by chemiluminescence with the Clarity Western ECL Substrate (Bio-Rad, Hercules, CA, United States) using the ChemiDoc MP Acquisition system (Bio-Rad, Hercules, CA, United States). The antibodies used in this study were Anti-SOD2 (ab137037), MitoProfile*®* Total OXPHOS Rodent WB Antibody Cocktail (ab110413) and Anti-β-Actin (sc-47778). For quantification, β-Actin was used as a loading control. The intensity of the individual bands was evaluated by ImageJ and normalized to the corresponding input control bands.

### 2.4. Oxygen consumption rate measurement

To determine mitochondrial respiratory capacity, we measured the oxygen consumption rate (OCR) using the Seahorse XF24 extracellular flux analyzer. We measured OCR following a previously established protocol designed for analyzing mitochondrial oxidative function in frozen tissues (18). Cardiac tissues were homogenized in a cold MAS buffer containing 70 mM sucrose, 220 mM mannitol, 5 mM KH_2_PO_4_, 5 mM MgCl_2_, 1 mM EGTA, and 2 mM HEPES, pH 7.4. The homogenates were centrifuged at 1,000 × g for 5 min at 4°C, and the supernatants were collected. The supernatants were further processed for mitochondrial isolation following the protocol by Osto et al. (18). Protein concentration was then determined using the Pierce BCA protein assay kit (Thermo Fisher Scientific). The samples containing 40 μg of total protein were loaded to each well of a Seahorse XF24 cell culture plate (Agilent).

### 2.5. Malondialdehyde determination

Blood samples were centrifuged and the plasma was isolated, aliquoted, and stored at −80°C until further analysis. Plasma level of malondialdehyde (MDA) was measured using the thiobarbituric acid-based procedure described by Wasowicz et al. (19). Briefly, 100 μl of plasma are added to the solution containing 29 mmol/L thiobarbituric acid in 8.75 mol/L acetic acid. Samples are heated for 60 min at 95°C and cooled down. The level of MDA was measured spectrophotometrically at 535 nm and expressed as µM concentrations using calibrating curves.

### 2.6. Morphology

The mice were sacrificed after 28 days of experimental protocol. Briefly, picrosirius red staining of cardiac cryosections (10 µm thick) was performed according to the standard protocol to visualize interstitial collagen. In stains for collagen content, we used a 0.1% solution of Sirius red in saturated aqueous solution of picric acid for 1 h at room temperature, followed by quickly washing in two changes of acidified water (0.5% acetic acid in water). Then, slides were dehydrated in ascending concentrations of ethanol and cleared in two stages in xylene. The slides were examined using light microscope equipped with a camera. The quantification of cardiac fibrosis was performed using ImageJ software.

### 2.7. Quantitative RT–PCR analysis

The expression level of genes was assessed using qRT-PCR. The total RNAs were isolated from cardiac muscle using RNeasy mini kit (Qiagen, Hilden, Germany). Total RNAs (300 ng) were reverse transcribed as previously described using Superscript II reverse transcriptase (Invitrogen, Waltham, MA, United States) (15). The sequences of the primers used are as follow and given in the 5′-3′ orientation: SOD2: GGACAAACCTGAGCCCTAAG (forward) and CAAAAGACCCAAAGTCACGC (reverse); Collagen type I: TGTGTGCGATGACGTGCAAT (forward) and GGGTCCCTCGACTCCTACA (reverse); Collagen type III: AAGGCGAATTCAAGGCTGAA (forward) and TGTGTTTAGTACAGCCATCCTCTAGAA (reverse); transforming growth factor beta-1 (TGFβ-1): GAGCCCGAAGCGGACTACTA (forward) and CACTGCTTCCCGAATGTCTGA (reverse); atrial natriuretic peptide (ANP): AGAGTGGGCAGAGACAGCAAA (forward) and AAGGCCAAGACGAGGAAGAAG (reverse); brain natriuretic peptide (BNP): GCACAAGATAGACCGGATCG (forward) and CCCAGGCAGAGTCAGAAAC (reverse); hypoxanthine phosphorybosyl transferase (HPRT): TGAAAGACTTGCTCGAGATGTCAT (forward) and TCCAGCAGGTCAGCAAAGAA (reverse). The validated PCR parameters were 5 min at 95°C followed by 40 cycles at 95°C for 15 s and 60°C for 1 min. The relative amount of target mRNA was calculated by the 2-^ΔΔCt^ method and expression of target gene was normalized to HPRT housekeeping gene expression.

### 2.8. Statistical analysis

Data were expressed as mean ± standard error of the mean (SEM). Statistical analysis between experimental groups was performed using unpaired Student’s t-test (GraphPad Prism version 9.3.1). A *P*-value of <0.05 was considered statistically significant.

## 3. Results

**Figure 1:**
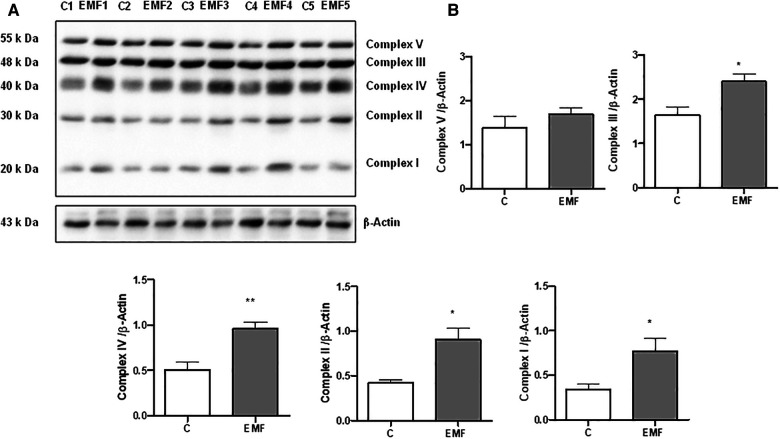
Myocardial expression of MitoProfile Total OXPHOS in mice exposed to 915 MHz EMFs for 28 days. (**A**) Representative Western blot image and (**B**) quantification of MitoProfile Total OXPHOS protein expression levels in control and EMF-exposed mice. The results present the mean ± SEM. *P < 0.05 vs. control, **P < 0.01 vs. control. EMFs, electromagnetic fields; OXPHOS, oxidative phosphorylation; SEM, standard error of the mean.

Defects in the mitochondrial OXPHOS system, a terminal biochemical pathway in energy production, result in deleterious, frequently multisystem disorders (3, 7). To determine changes in mitochondrial energy metabolism in conditions of prolonged electromagnetic stress, we examined the effects of EMFs on the expression level of OXPHOS proteins after 28 days. Total protein extracts from heart tissues of mice subjected to 915 MHz frequency EMFs were subjected to immunoblotting with the Total OXPHOS Rodent Antibody Cocktail kit to detect all five complexes simultaneously (Figure 1). The densitometric quantification of the bands from immunoblots demonstrated a significant increase in the expression level of Complex I and II subunits in EMF-exposed mice compared to control animals. Moreover, myocardial expression of Complex III and Complex IV subunits increased significantly in mice subjected to electromagnetic stress (Figure 1). However, the cardiac level of the α-subunit of ATP synthase (Complex V) remained stable among groups (Figure 1).

**Figure 2:**
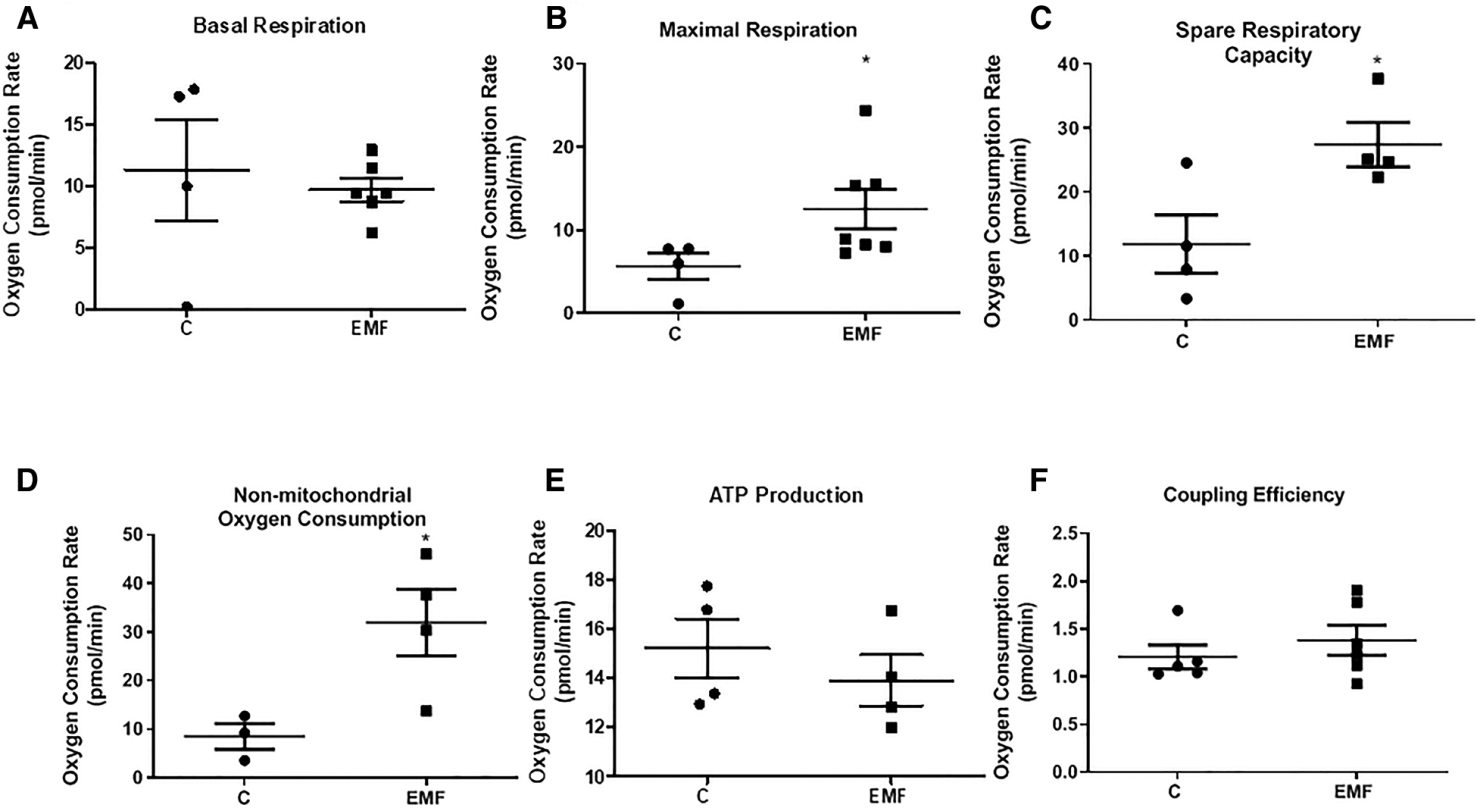
Mitochondrial respiratory function in mice exposed to 915 MHz EMFs for 28 days. (**A**) Basal respiration, (**B**) maximal respiration, (**C**) spare respiratory capacity, (**D**) non-mitochondrial oxygen consumption, (**E**) ATP production, and (**F**) coupling efficiency in control and EMF-exposed mice. The results present the mean ± SEM. *P < 0.05 vs. control. EMFs, electromagnetic fields; ATP, adenosine triphosphate; SEM, standard error of the mean.

To determine if the observed modifications in protein expression translate into altered mitochondrial function, we next examined mitochondrial respiration in isolated mitochondria obtained from control and EMF-exposed mouse hearts using the Agilent Seahorse XF Cell Mito Stress test. As shown in Figure 2, no significant changes in mitochondrial basal respiration were noted between control and EMF-exposed groups; however, mitochondrial maximal respiration and the spare respiratory capacity in EMF-challenged mitochondria were significantly elevated compared to the control group. Because oxygen consumption can be non-mitochondrial, respiration specific to the cellular non-mitochondrial component was assessed by inhibiting mitochondrial Complex I and Complex III with rotenone and antimycin A. As shown in Figure 2, the electromagnetic stress also significantly increased the non-mitochondrial oxygen consumption compared to the control (Figure 2). However, ATP production and coupling efficiency were unchanged between control and EMF-challenged mice (Figure 2). Representative plot showing OCRs of cardiac mitochondria isolated from control and EMF-exposed mice is presented in Supplementary Figure S1.

**Figure 3:**
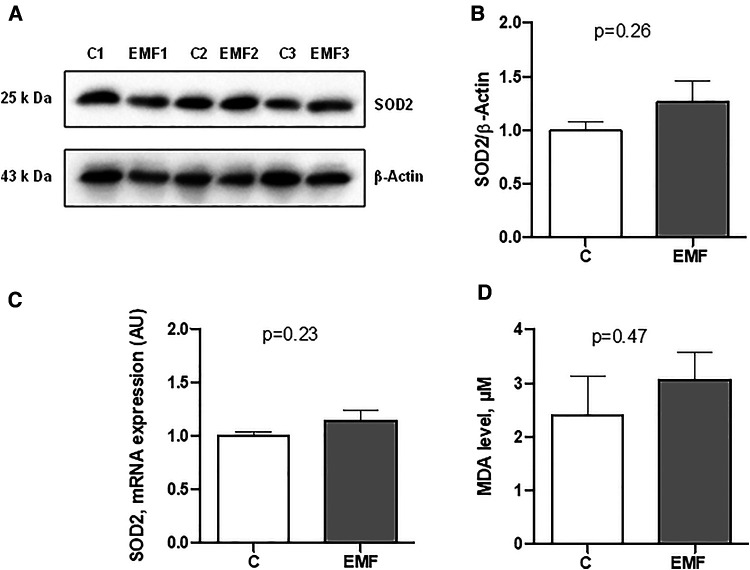
Myocardial expression of SOD2 in mice exposed to 915 MHz EMFs for 28 days. (**A**) Representative Western blot image, (**B**) quantification of SOD2 protein expression levels, (**C**) qRT-PCR analysis of SOD2 mRNA expression level and (**D**) plasma MDA level in control and EMF-exposed mice. The results present the mean ± SEM. EMFs, electromagnetic fields; SOD2, superoxide dismutase 2; qRT-PCR, quantitative polymerase chain reaction; MDA, malondialdehyde; SEM, standard error of the mean.

To elucidate whether the elevated state respiration in EMF-challenged cardiac tissue was associated with the alterations of antioxidant system, we assessed gene and protein expressions of SOD2, a key component of the mitochondrial antioxidant defense. As shown in Figures 3A–C, myocardial expression levels of mRNA and SOD2 protein expression were unchanged between control and EMF-exposed mice.

We next measured the plasma level of MDA, a lipid peroxidation product that is used for the evaluation of oxidative stress (20, 21). As shown in Figure 3D, no statistical difference was observed in terms of the MDA levels in EMF-exposed mice compared to the controls.

**Figure 4:**
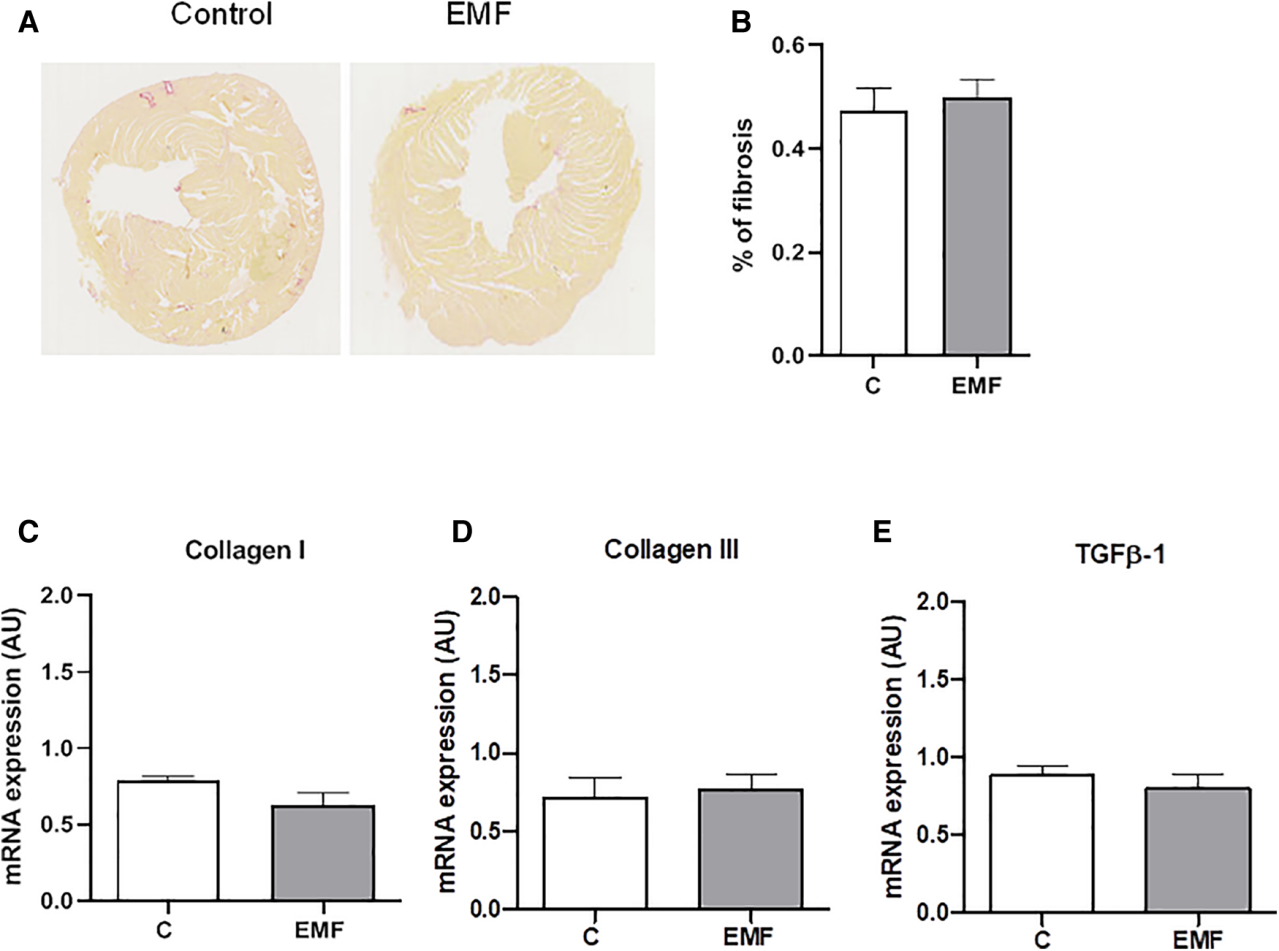
Prolonged effect of EMFs on cardiac collagen content in mice subjected to 915 MHz EMFs for 28 days. (**A**) Representative image of Sirius red-stained cardiac sections, (**B**) quantification of (**A**), (**C**) qRT-PCR analysis of mRNA expression levels of Collagen type I, (**D**) Collagen type III, and (**E**) TGFβ-1. The results present the mean ± SEM. EMFs, electromagnetic fields; qRT-PCR, quantitative polymerase chain reaction; TGFβ-1, transforming growth factor beta-1; SEM, standard error of the mean.

Mitochondrial metabolism and structural tissue integrity are critical links in response to stressors. We next examined the total content of collagen, the most abundant component of extracellular matrix (ECM) in the heart. As shown in Figure 4, no statistically significant differences in the total collagen level were observed in the cardiac tissue between the control and EMF-exposed groups.

To confirm the histomorphological observation, the gene expression levels of Collagen I and Collagen III, the major structural components of the myocardial ECM, were examined via qRT-PCR. As shown in Figure 4, mRNA expression of Collagen types I and III were not significantly different between the control and EMF-exposed groups. We also found that mRNA expression of TGFβ-1, a cardiac fibrotic factor, was comparable among control and EMF-challenged mice.

**Figure 5:**
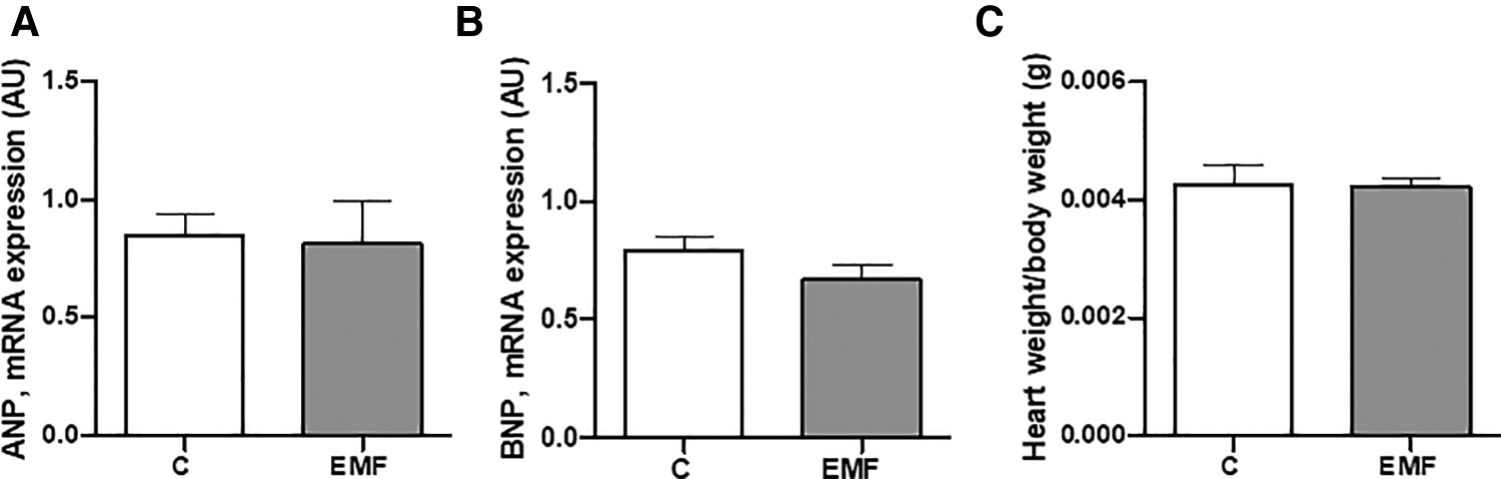
The effects of prolonged exposure to EMFs on structural integrity of cardiac tissue. qRT-PCR analysis of mRNA expression level of ANP (**A**) and BNP (**B**), and (**C**) ratio of heart weight to body weight in control group and in mice subjected to 915 MHz EMFs for 28 days. The results present the mean ± SEM. EMFs, electromagnetic fields; qRT-PCR, quantitative polymerase chain reaction; ANP, atrial natriuretic peptide; BNP, brain natriuretic peptide; SEM, standard error of the mean.

To examine whether EMFs could cause the abnormalities of left ventricular function and hypertrophy in mice, we next measured the level of ANP and BNP. As shown in Figures 5A,B, no statistically significant differences in ANP and BNP levels were observed in the cardiac tissue between the control and EMF-challenged groups. In addition, the average heart weight/body weight ratio was not significantly different between control and EMF-exposed mice (Figure 5C).

## 4. Discussion

Mitochondrial metabolic reprogramming is a distinctive characteristic of cardiac cells and occurs as a consequence of adaptation or maladaptation to the environmental stress (22, 23). In a healthy heart, mitochondrial oxidative metabolism provides ATP and precursors for macromolecular biosynthesis to meet the energy requirements for cardiac cell survival (24, 25). The predominant mechanisms of cardiac damage are the defects in OXPHOS capacity and oxidative stress. Metabolic reprogramming enables cardiac cells to adapt and orchestrate oxidative stress responses (26–28). The precise picture of how electromagnetic stress calibrates metabolic changes in cardiac tissue via OXPHOS system is obscure. Here, we demonstrate for the first time that prolonged exposure to 915 MHz EMFs for 28 days affects the respiratory capacity of mitochondria without compromising the structural integrity of the heart. These findings provide new insights into the essential role of mitochondrial respiration in cardiac adaptation to prolonged electromagnetic stress.

There is a long-running debate on whether EMFs impact mitochondrial bioenergetics in living systems. In cardiac tissue, mitochondrial oxidative metabolism is the principal source of energy, which underscores the essential role of mitochondria in heart’s performance (26). Mitochondria compose 30% of cardiomyocyte volume and supply cardiac energy status through OXPHOS machinery (29). A healthy heart is metabolically flexible, as it is able to shift between OXPHOS components to provide adequate ATP generation in response to stress (30–32). However, defects in the OXPHOS system can modify metabolic homeostasis in cardiac cells and contribute to heart failure (33–35). In the present study, analysis of the mitochondrial OXPHOS system in EMF-challenged hearts after 28 days pointed to increased protein level expression of Complex I, the largest component of the respiratory chain. Mitochondrial Complex I subunit is capital for maintaining the functional integrity of the respiratory complexes and to provide efficient transfer of electrons between electron transport chain (ETC) complexes. In a failing heart, mitochondrial respiratory Complex I is highly sensitive to structural and functional damage, which can contribute to postischemic mitochondrial ETC disorders, decline in respiratory function, and energy supply deficits (36). We also demonstrated that cardiac Complex II and III were upregulated in the group of mice subjected to EMFs for 28 days. Large quantities of evidence state the respiratory Complex II subunit as a fondement and calibrator of mitochondrial ROS. Both functional loss of Complex II and pharmacological inhibition can result in ROS generation in cardiac cells, with a relevant impact on the development of pathophysiological manifestations. Respiratory Complexes I, II, and III are the capital sites for ROS generation (37). Mitochondrial Complex I inhibition by rotenone can trigger ROS generation in submitochondrial modifications. Several studies reported that oxidation of either Complex I or Complex II substrates in the presence of Complex III suppression with antimycin A favors ROS formation (38, 39). In myocardial infarction, rotenone reduces ROS overproduction and limits mitochondrial damage in the isolated rat heart (38). Recent evidence points to the respiratory Complex II as a source and calibrator of mitochondrial ROS. Both pharmacological inhibition and functional loss of Complex II can lead to ROS formation in cardiac cells, with a relevant impact on the development of pathophysiological conditions including heart disease (37, 40). In the cardiovascular system, Complex II can contribute notably to ROS production both directly and indirectly (via reverse electron transfer), with physiological and pathological impacts (37, 41). Abnormal mitochondrial ROS generation is involved in cardiac ischemia-reperfusion (I/R) injury, and Complex II inhibitors exert protective effects in I/R models by suppressing reverse electron transport (RET) (42, 43). We found that in mice exposed to EMFs for 28 days, the cardiac expression level of Complex IV was upregulated, while Complex V level expression remained relatively stable. In OXPHOS machinery, Complex IV catalyzes the mitochondrial ETC and transformation of oxygen to water, linked to proton translocation (44–46). Recent studies suggest that mitochondrial Complex IV dysfunction drives cell adaptive signaling and metabolic perturbations (47). In cancer cells, dysregulation of mitochondrial Complex IV may stimulate the metastatic potential and reflect the positive correlation to metastatic spread of cancer (47–49). Our findings suggest that prolonged exposure to EMFs alters mitochondrial oxidative metabolism through modulating the OXPHOS system in the heart. Furthermore, using the Seahorse Extracellular Flux Analyzer, we examined mitochondrial respiration in isolated mitochondria obtained from control and EMF-exposed mouse hearts. We found that prolonged electromagnetic stress increases mitochondrial maximal respiration and spare respiratory capacity. In addition, we demonstrated the electromagnetic stress also significantly enhances non-mitochondrial oxygen consumption.

The increased mitochondrial respiration in conditions of electromagnetic stress may be an adaptive response on the cardiac level, illustrating a fundamental link between environmental influence and myocardial energy metabolism. Interestingly, cardiac adoption of metabolic upregulation of Complexes I, II, III, and IV was uncoupled to cardiac expression of SOD2, a first-line component of mitochondrial antioxidant defense against superoxide produced by respiration. Indeed, we did not observe significant differences in SOD2 expression among control and EMF-exposed mice for 28 days. However, further studies are needed to evaluate the total myocardial antioxidant potential in cardiac tissue under prolonged electromagnetic stress.

In addition, we evaluated the effect of prolonged exposition to EMFs on the plasma MDA level, one of known indices of oxidative stress. Free oxygen radicals enhance lipid peroxidation, and MDA is a lipid peroxidation product that is used for the evaluation of oxidative stress (50, 51). In our experiments, no significant difference was found between plasma MDA level in EMF-exposed and control groups suggesting that prolonged exposure to electromagnetic stress does not alter lipid peroxidation.

To address the structural and functional state of left ventricles, we evaluated the myocardial level of ANP and BNP in mice subjected to prolonged electromagnetic stress. Currently, ANP and BNP are widely used as significant indicators for the cardiac dysfunction and clinical diagnosis of heart failure in clinical medicine (52, 53). Our findings point to a constant level of ANP and BNP in EMF-challenged hearts isolated from mice subjected to EMFs for 28 days, suggesting that prolonged electromagnetic stress does not induce cardiac abnormalities. We have recently demonstrated that short-term exposure to EMF for 48 and 72 h in cardiac tissues did not alter the cardiac structural integrity in terms of collagen deposition, necrotic myofibers, and cardiomyocyte size (15). In the present study, we also evaluated the cardiac collagen level, the main structural element forming the ECM. Analysis of cardiac sections from EMF-exposed mice exposed for 28 days demonstrated that collagen content was not affected suggesting that prolonged electromagnetic stress did not induce cardiac fibrosis. Several studies reported abnormalities in collagen synthesis and deposition under electromagnetic stress (54, 55). Soda et al. demonstrated that exposure to low-frequency EMFs (3 mT, 60 Hz) increases the collagen synthesis in mouse osteoblasts—like MC3T3-E1 cells (55). A previous study has demonstrated that EMFs can accelerate collagen production in bone and cartilage due to effects of K, Ca, and Mg ion transport (54). In conditions of myofibroblast activation linked to the diabetic wound healing, Choi et al. reported elevated collagen fibers in response to electromagnetic stress (56). These observations clearly suggest a wide variability of EMF-related effects on the structural components of ECM. Further research into the mechanisms that regulate ECM integrity in cardiac tissue under electromagnetic environment is required.

## 5. Conclusion

The ability of cardiac cells to adjust the mitochondrial oxidative metabolism in response to environmental stress is a well-recognized signature of the cardiovascular system. Persistent metabolic changes may result in tissue abnormalities or may trigger cell maladaptive responses. Our study points at notable differences between control and EMF-challenged mice hearts at the level of mitochondrial respiration after prolonged EMF exposure. As the mouse heart imitates the mammalian cardiovascular phenotype, our data suggest that metabolic oxidative status changes dynamically in response to EMFs in the myocardium, challenging our quest to better understand cardiac cell biology under conditions of electromagnetic environment.

## Data Availability

The raw data supporting the conclusions of this article will be made available by the authors, without undue reservation.
