# Supplementary figures and images for: Myocardial capacity of mitochondrial oxidative phosphorylation in response to prolonged electromagnetic stress
Source: Front Cardiovasc Med. 2023 Jun 7;10:1205893. doi: 10.3389/fcvm.2023.1205893 (PMC10282661; doi:10.3389/fcvm.2023.1205893)

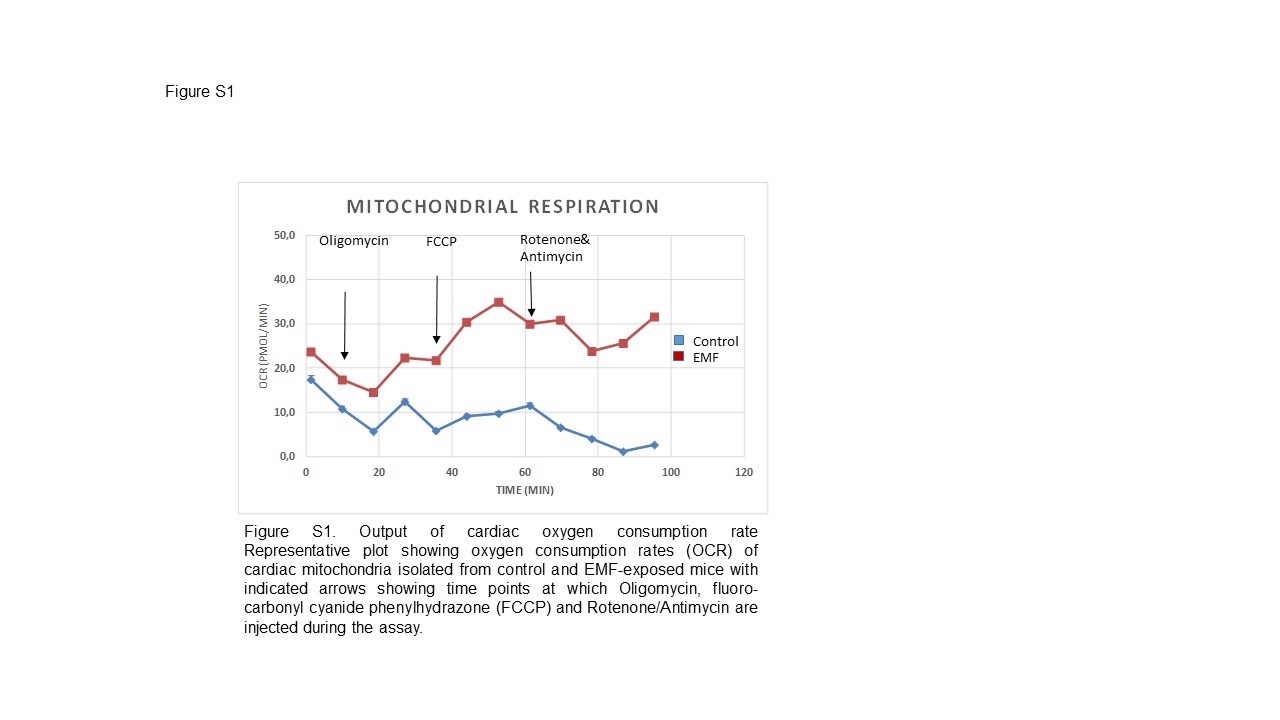

Supplement: Supplementary file 1 [file Image1.jpeg]
